# β1-integrin-dependent migration of microglia in response to neuron-released α-synuclein

**DOI:** 10.1038/emm.2014.6

**Published:** 2014-04-18

**Authors:** Changyoun Kim, Eun-Deok Cho, Hyung-Koo Kim, Sungyong You, He-Jin Lee, Daehee Hwang, Seung-Jae Lee

**Affiliations:** 1 Department of Biomedical Science and Technology, Konkuk University, Seoul, Korea; 2 IBST, Konkuk University, Seoul, Korea; 3 Department of Anatomy, School of Medicine, Konkuk University, Seoul, Korea; 4 Samuel Oschin Comprehensive Cancer Institute, Cedars-Sinai Medical Center, Los Angeles, CA, USA; 5 School of Interdisciplinary Bioscience and Bioengineering and Department of Chemical Engineering, POSTECH, Pohang, Kyoungbuk, Korea

**Keywords:** α-synuclein, β1-integrin, microglial migration, neuroinflammation, Parkinson's disease

## Abstract

Chronic neuroinflammation is an integral pathological feature of major neurodegenerative diseases. The recruitment of microglia to affected brain regions and the activation of these cells are the major events leading to disease-associated neuroinflammation. In a previous study, we showed that neuron-released α-synuclein can activate microglia through activating the Toll-like receptor 2 (TLR2) pathway, resulting in proinflammatory responses. However, it is not clear whether other signaling pathways are involved in the migration and activation of microglia in response to neuron-released α-synuclein. In the current study, we demonstrated that TLR2 activation is not sufficient for all of the changes manifested by microglia in response to neuron-released α-synuclein. Specifically, the migration of and morphological changes in microglia, triggered by neuron-released α-synuclein, did not require the activation of TLR2, whereas increased proliferation and production of cytokines were strictly under the control of TLR2. Construction of a hypothetical signaling network using computational tools and experimental validation with various peptide inhibitors showed that β1-integrin was necessary for both the morphological changes and the migration. However, neither proliferation nor cytokine production by microglia was dependent on the activation of β1-integrin. These results suggest that β1-integrin signaling is specifically responsible for the recruitment of microglia to the disease-affected brain regions, where neurons most likely release relatively high levels of α-synuclein.

## Introduction

Parkinson's disease (PD) is an age-related neurodegenerative disease characterized by motor and non-motor symptoms.^1^ Pathologically, PD is characterized by the loss of dopamine neurons in the substantia nigra pars compacta and is associated with abnormal protein accumulation in forms known as Lewy bodies (LBs) and Lewy neurites.^2^ LBs and Lewy neurites are composed of various proteins; however, the amyloid fibril form of α-synuclein is the predominant component.^3^ PD is a multifactorial disorder; however, a large body of evidence has suggested that α-synuclein has important roles in the onset and progression of the disease.^4, 5^

Although α-synuclein is a typical neuronal cytosolic protein, some α-synuclein is released from neurons^6^ and is present in body fluids such as the cerebrospinal fluid, serum and brain interstitial fluid.^7, 8^ Extracellular α-synuclein is transferred to neighboring neurons and astrocytes, promoting abnormal accumulation of α-synuclein, which induces cell death in the neurons^9, 10^ and an inflammatory response in the astrocytes.^11^

Neuroinflammation is a key feature of most neurodegenerative diseases, and microglia have critical roles in the process of neuroinflammation.^12^ Several studies have suggested the involvement of neuroinflammation in the development and progression of these diseases, especially in PD.^13, 14, 15^ For example, activated microglia have been found in the substantia nigra of PD patients, and degeneration of dopaminergic neurons has been inhibited by the administration of an anti-inflammatory drug in a toxicant-induced PD animal model.^16^ In addition, several epidemiological studies have suggested that anti-inflammatory drugs, especially non-steroidal anti-inflammatory drugs, reduce the risk of PD.^15^ Therefore, it is likely that neuroinflammation not only is a pathological epiphenomenon but also may have critical roles in the disease progression.

Microglia are a type of resident immune cells in the brain, and they contribute to the innate immune response.^17^ Microglia can be activated by various types of stimuli, including inflammatory stimuli, brain injury and ischemia.^18^ Upon stimulation, microglia undergo a series of activation processes, including morphological changes, proliferation, increased production of intracellular reactive oxygen species and proinflammatory cytokine gene induction. Chronic activation of microglia can lead to neuronal damage.^13^

Microglial cells exposed to neuron-released α-synuclein exhibited features of activated microglia, including a morphological transition from a ramified to an ameboid shape, increased proliferation and production of proinflammatory factors such as inflammatory cytokines, reactive oxygen species and nitric oxide.^14^ In our previous study, we demonstrated that some of these changes, such as the induction of cytokines, are mediated by the activation of toll-like receptor 2 (TLR2).^14^ However, it is not clear whether activation of TLR2 signaling is sufficient to elicit all of the features of microglial activation that are triggered by neuron-released α-synuclein.

In the current study, we demonstrate that TLR2 activation is not sufficient for all of the changes manifested by microglia in response to neuron-released α-synuclein. Specifically, the changes in microglial migration and morphology that are triggered by neuron-released α-synuclein required β1-integrin, whereas the increased proliferation and increased production of cytokines are strictly under the control of TLR2.

## Materials and methods

### Animals

Sprague–Dawley rats and C57BL/6 mice were purchased from Samtako (Osan, Korea). TLR2-deficient mice were obtained from Oriental Bioservice (Kyoto, Japan).^19^ The animal use protocols in this study were approved by Konkuk University's Animal Care and Use Committee.

### Materials

The peptides 4N1K (KRFYVVMWKK) and RHD peptide were purchased from Bachem (Bubendorf, Switzerland). WRW4 was obtained from Tocris Bioscience (Ellisville, MO, USA). GRGDS and SDGRG peptides were purchased from Sigma Aldrich (St. Louis, MO, USA).

### Cell culture

Human SH-SY5Y neuroblastoma cells and primary microglia (rat and mouse) were maintained according to a previously described protocol.^14^

### Preparation of conditioned medium

SH-SY5Y neuroblastoma cells were differentiated in the presence of retinoic acid for 5 days and then infected with adenoviral vectors expressing α-synuclein (adeno/α-syn) and LacZ (adeno/lacZ) at a multiplicity of infection of 100. After 2 days of incubation, these cells were incubated in serum-free Dulbecco's modified Eagle's medium for 18 h. The culture supernatants were centrifuged at 1000 × *g* for 10 min and subsequently at 10 000 × *g* for 10 min. The concentration of α-synuclein in the medium was on average 1.06±0.371 μg ml^−1^ (s.e.m.).^14^ The supernatants were concentrated using 10 000 molecular weight cutoff centrifugal filters (Millipore, County Cork, Ireland).

### Morphological analysis of microglia

Primary rat or mouse microglia were cultured in poly-D-lysine-coated 12-well culture plates. On the following day, cells were treated with either conditioned medium or lipopolysaccharide for 24 h. The morphology of microglia (ameboid/ramified) was analyzed in 10 randomly chosen areas for each independent experiment, and the mean values were obtained.

### Reverse transcription polymerase chain reaction (RT-PCR)

Microglia were treated with conditioned medium for 6 h, and the total RNA was then extracted from the cells using an RNeasy Mini kit (Qiagen, Hilden, Germany), according to the manufacturer's protocol. Equal amounts of total RNA were reverse-transcribed using an iScript complementary DNA synthesis kit (Bio-Rad, Hercules, CA, USA). The complementary DNA products were amplified with PCR using specific primers and temperature cycles, as indicated (Table 1).

### Reconstruction of integrin network model

To reconstruct the integrin network model, we used 2009 differentially expressed genes (DEGs) (877 DEGs at 6 h only, 797 DEGs at 24 h only and 335 DEGs common to both time points) that were identified in our previous study (GSE 26532).^14^ Functional enrichment analysis of upregulated genes was performed using DAVID to identify the cellular processes and pathways governed by α-synuclein conditioned medium (αSCM) at 6 and 24 h. We selected a subset of genes associated with focal adhesion and the leukocyte transendothelial migration pathway. This subset contained motifs indicative of well-defined signaling downstream of integrins and protein–protein interactions. Using these criteria, genes were selected from among all upregulated genes listed in the KEGG pathway database.^20^ The selected genes and their interactions were visualized using Cytoscape.^21^ The network nodes were arranged according to the pathway map of the corresponding genes. The node and border colors indicate mRNA abundance in αSCM-exposed primary rat microglia that is higher (red) or lower (green) than in those exposed to LacZ-conditioned medium (LZCM) at 6 and 24 h, respectively. The resulting network describes the putative integrin-dependent downstream signaling regulated by α-synuclein in primary rat microglia cultured in conditioned medium.

### Cell viability assay

Rat primary microglia were seeded into dark 96-well cell culture plates coated with PDL. They were then treated with conditioned medium and antagonists. After 24 h of incubation, the viability of the attached cells in each culture plate was determined using a CyQUANT cell proliferation assay kit (Invitrogen, Carlsbad, CA, USA).

### Wound-healing assay

Primary rat microglia were seeded into PDL-coated 12-well cell culture plates. The following day, a 200-μl tip was used to scratch the surface of the cell culture, and the recovery width of the scratched surface was then measured at nine marked sites.^22, 23^ Microglia were pre-incubated with antagonists for 30 min before the addition of the conditioned medium to the culture medium. After a further 18-h incubation, the recovery width of the scratched surface was re-measured at the nine marked sites for each independent experiment, and the mean values were calculated.

### Statistical analysis

InStat (GraphPad Software, San Diego, CA, USA) was used for all statistical analysis. All data are presented as means±s.e.m. All data were analyzed for statistical significance by using unpaired *t*-tests.

## Results

### TLR2-dependent and -independent microglial responses to neuron-released α-synuclein

**Figure 1:**
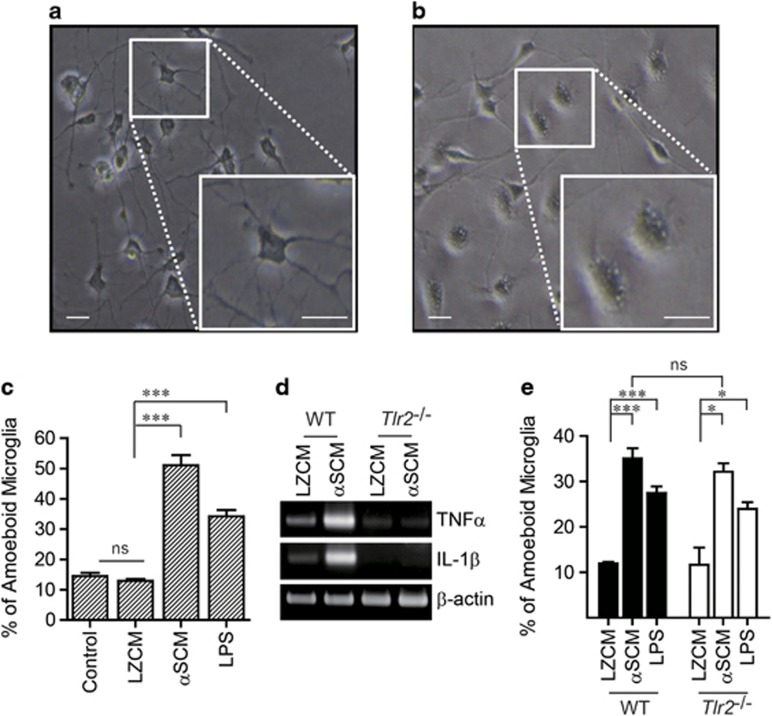
TLR2-dependent and -independent microglial activation by neuron-released α-synuclein. Rat and mouse primary microglia were treated with LZCM, αSCM or lipopolysaccharide (LPS) (1 μg ml^−1^, as a positive control) for 24 h (**a**–**c**) or 6 h (**d**). (**a**–**c**) αSCM- or LPS-induced morphological changes in rat primary microglia: from the resting shape (**a**) to an ameboid shape (**b**). (**c**) The percentage of ameboid microglia was determined by microglial morphology analysis (*n*=3). (**d**) Wild-type and TLR2-deficient mouse microglia were treated with conditioned medium for 6 h, and total mRNA was then extracted and reverse-transcribed. The expression of cytokine genes (tumor necrosis factor α (TNFα) and interleukin (IL)-1β) was determined using conventional RT-PCR. (**e**) Murine primary microglia from wild-type and TLR2-deficient mice were treated with LZCM, αSCM or LPS for 24 h. The percentage of ameboid microglia was determined by microglial morphology analysis (*n*=3). Morphology analysis data were analyzed using an unpaired *t*-test. Scale bars, 10 μm. Error bars represent the s.e.m. *P<0.05; ^***^P<0.001. ‘*n*' represents the number of independent experiments.

Our previous study demonstrated a role for TLR2 in at least some aspects of neuron-released α-synuclein-induced microglial activation. To extend our understanding of α-synuclein-induced microglial activation, we obtained conditioned medium from differentiated SH-SY5Y cells overexpressing either α-synuclein or LacZ (αSCM, LZCM), as well as mouse or rat primary microglia treated with each of these conditioned media. Upon exposure to αSCM, microglia underwent morphological changes from resting to ameboid shapes (Figures 1a–c). Treatment with lipopolysaccharide, an activator of microglia, also induced morphological changes in the microglia (Figure 1c). In contrast, morphological changes were absent in LZCM-exposed microglia (Figures 1a and c). To analyze cytokine gene induction, mouse primary microglia were treated with αSCM for 6 h, and proinflammatory cytokine gene expression was investigated by RT-PCR. The expression of interleukin-1β and tumor necrosis factor (TNF)α increased in response to treatment with αSCM. These changes were dependent on TLR2 because TLR2 gene depletion eliminated cytokine gene induction (Figure 1d). In contrast to cytokine gene induction, there was no significant difference between the normal microglia and the TLR2-deficient microglia in the extent of morphological changes upon exposure to neuronal cell-released α-synuclein (Figure 1e). These results suggest the existence of two distinct regulatory mechanisms for microglial responses to neuronal cell-released α-synuclein: a TLR2-dependent mechanism and a TLR2-independent mechanism.

### Construction of a hypothetical network for neuron-released α-synuclein-induced morphological changes in microglia

**Figure 2:**
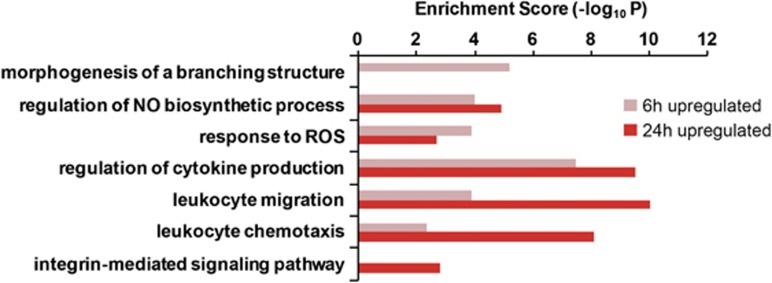
Gene expression profiling analysis of microglia exposed to αSCM. The enriched KEGG pathways were identified from upregulated genes in αSCM-treated rat primary microglia, and the enrichment scores are displayed for each pathway.

**Figure 3:**
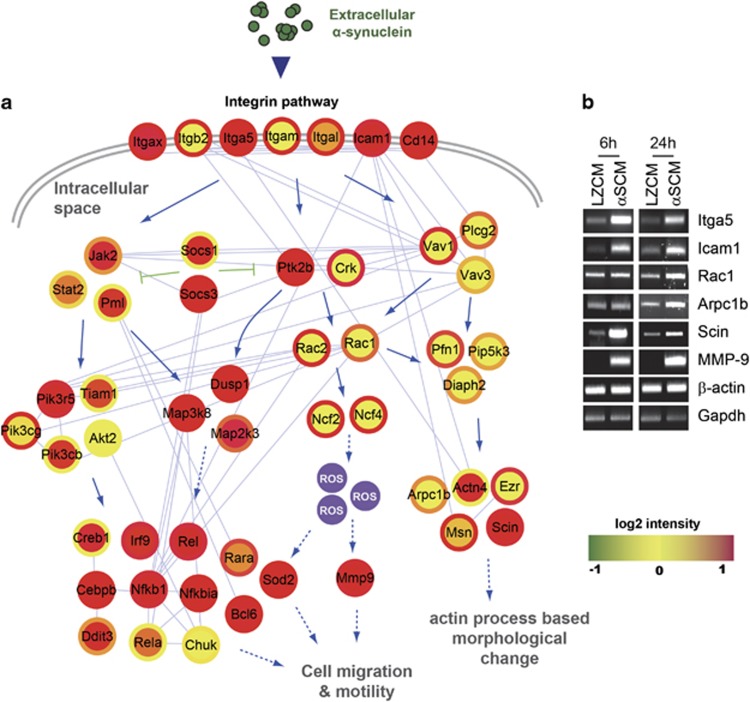
Hypothetical signaling network related to morphological changes and migration of microglia exposed to neuron-released α-synuclein. Rat primary microglia were treated with LZCM or αSCM. (**a**) To analyze alterations in gene expression, total mRNA was extracted from microglia and submitted to microarray analysis. DEGs were detected at two different time points (6 and 24 h). A hypothetical signaling network was constructed using DEGs from αSCM-exposed microglia (inner circle, 6 h; outer circle, 24 h). (**b**) Changes in the expression of the identified genes in the network were determined using conventional RT-PCR.

To investigate the mechanism underlying the morphological changes in microglia in response to αSCM, we analyzed, in detail, the gene expression profile data that we obtained in the previous study (GSE 26532).^14^ The KEGG pathway enrichment analysis of upregulated genes suggested that the integrin signaling pathway would be activated as a late response, with high statistical significance (Figure 2 and Table 2). This is in addition to the activation of the TLR2 signaling pathway, which was extensively characterized in our previous study.^14^ On the basis of these data, we postulated that the integrin signaling pathway might represent another key mechanism of microglial response to neuron-released α-synuclein, especially the mechanism controlling morphological changes. Using DEG data and protein–protein interaction data derived from a public database (NCBI GEO), we constructed a hypothetical signaling network model for the integrin signaling triggered by αSCM (Figure 3a). The network model suggested that the integrin signaling pathway was responsible for microglial morphological changes. Induction of gene expression in the integrin signaling cascade was verified using RT-PCR in microglia after exposure to αSCM (Figure 3b). Furthermore, the hypothetical network model suggested that activation of the integrin signaling resulted in increased cell motility in addition to morphological changes, both of which involve extensive actin rearrangement (Figure 3a).

### Role of β1-integrin in αSCM-induced microglial morphological changes

**Figure 4:**
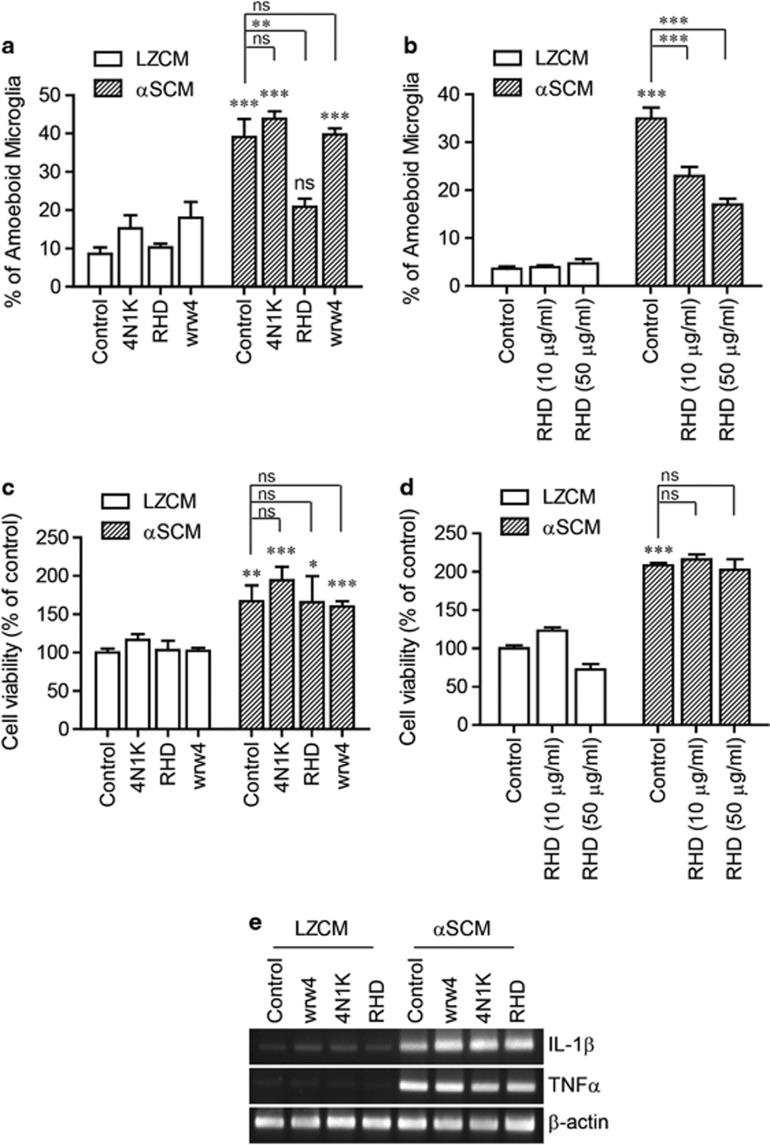
Integrin-dependent morphological changes of microglia by neuron-released α-synuclein. (**a**, **c**, **e**) Rat primary microglia were pretreated with 4N1K (100 μg ml^−1^), RHD (20 μg ml^−1^) and WRW4 (20 μg ml^−1^) for 30 min and then incubated with a conditioned medium for 24 h (**a**, **c**) or 6 h (**e**). (**b**, **d**) Rat primary microglia were pretreated with the indicated concentration of RHD for 30 min and then exposed to a conditioned medium for 24 h. (**a**, **b**) The percentage of ameboid microglia was determined by microglial morphology analysis (*n*=3). (**c**, **d**) Cell proliferation was determined by measuring cellular nucleic acid contents (*n*=3). (**e**) The expression levels of cytokine genes (TNFα and IL-1β) were determined using conventional RT-PCR. All data were analyzed using unpaired *t*-tests. Error bars represent the s.e.m. *P<0.05; ^**^P<0.01; ^***^P<0.001. ‘*n*' represents the number of independent experiments.

To determine the role of integrins in microglial responses to neuron-released α-synuclein, we tested the effects of functional peptide antagonists on various cell surface receptors. Primary rat microglia were pre-incubated with antagonists for 30 min before the addition of αSCM, and the morphological changes of the microglia were analyzed after 24 h. αSCM-induced morphological changes were significantly inhibited by the RHD peptide, a β1-integrin-interacting peptide from amyloid beta^24, 25^ (Figure 4a). However, other peptide antagonists, 4N1K (an antagonist of CD47) and WRW4 (an antagonist of formyl peptide chemotactic receptor-like 1), did not affect the morphological changes of microglia by αSCM (Figure 4a). The effects of RHD on the αSCM-induced morphological changes were dose-dependent, further validating the role of integrins (Figure 4b). Conversely, αSCM-induced microglial proliferation and cytokine gene expression were not affected by pre-treatment with RHD (Figures 4c–e). These results suggest that the integrin signaling pathway is specifically responsible for the morphological changes but not for cell proliferation or cytokine gene induction.

### β1-integrin-dependent morphological changes and migration of microglia by neuronal cell-released α-synuclein

**Figure 5:**
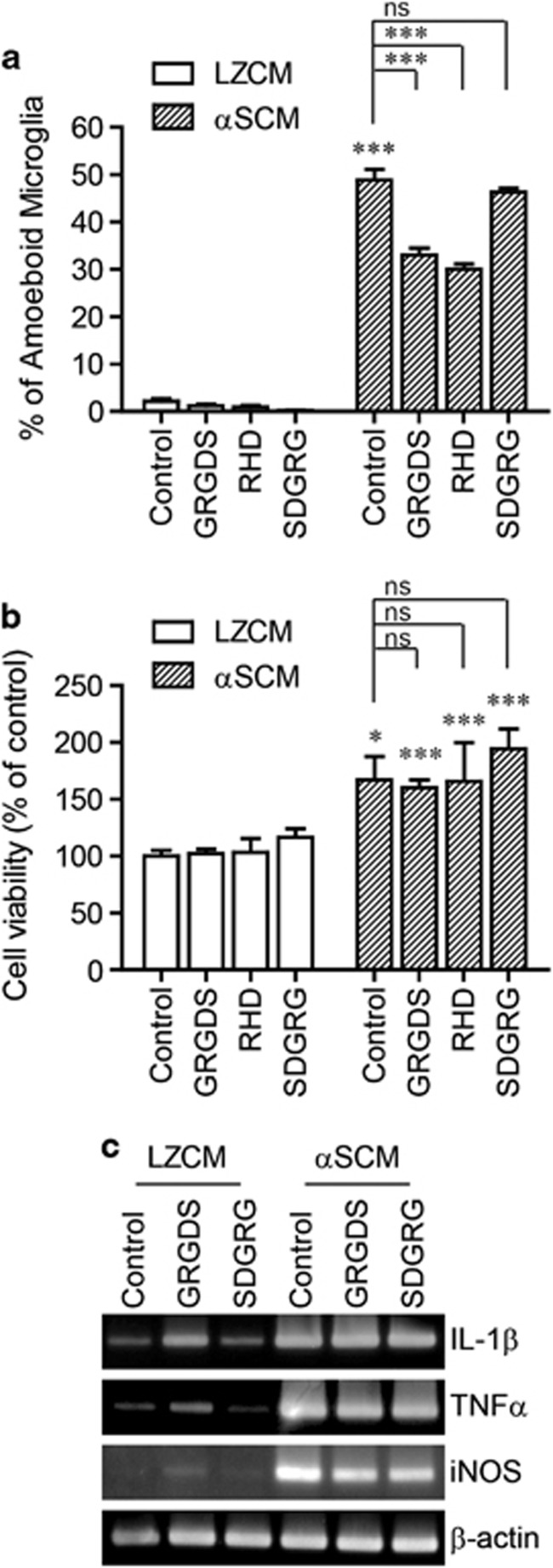
β1-Integrin-dependent morphological changes in microglia in response to neuron-released α-synuclein. Rat primary microglia were pretreated with GRGDS (20 μg ml^−1^), RHD (20 μg ml^−1^) or SDGRG (20 μg ml^−1^) for 30 min and then incubated with αSCM for 24 h (**a**, **b**) or 6 h (**c**). (**a**) Microglial morphology analysis (*n*=3). (**b**) Cell proliferation assay (*n*=3). (**c**) The expression levels of TNFα, IL-1β and iNOS were determined using conventional RT-PCR. All data were analyzed using unpaired *t*-tests. Error bars represent the s.e.m. *P<0.05; ^***^P<0.001. ‘*n*' represents the number of independent experiments.

To validate the role of β1-integrin in αSCM-induced morphological changes of microglia, we used a specific antagonist peptide of β1-integrin, GRGDS. As expected, pre-incubation with GRGDS inhibited αSCM-induced morphological changes in the microglia (Figure 5a). However, SDGRG, a control peptide with the reverse sequence of GRGDS, did not affect the morphological changes in the microglia (Figure 5a). Consistent with the data obtained with the RHD peptide, αSCM-induced microglial proliferation (Figure 5b) and cytokine gene induction (Figure 5c) were not altered by GRGDS pre-incubation.

**Figure 6:**
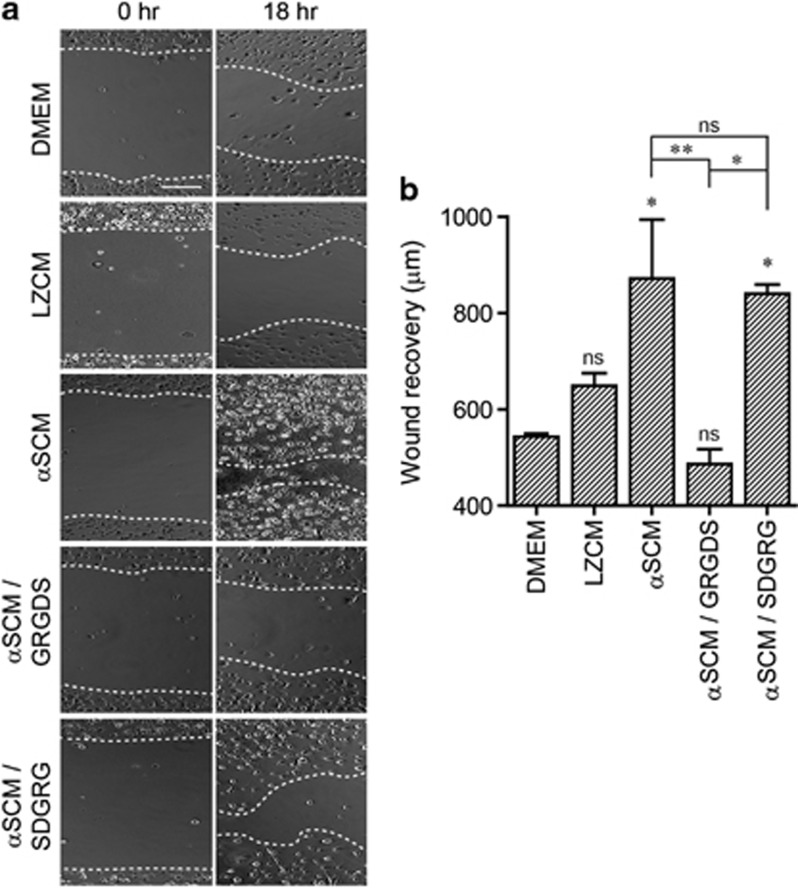
β1-Integrin-dependent migration of microglia in response to neuron-released α-synuclein. The migration of rat primary microglia was measured using a wound-healing assay. Rat primary microglia were pretreated with Dulbecco's modified Eagle's medium, GRGDS (20 μg ml^−1^) or SDGRG (20 μg ml^−1^) for 30 min and then incubated with αSCM. After an 18-h incubation, the widths of the marked wounds were measured using a light microscope. (**a**) Representative images of microglia in the wound-healing assay. (**b**) Migration of microglia is expressed as the length between the wound boundaries (*n*=3). Data were analyzed using unpaired *t*-tests. Error bars represent the s.e.m. *P<0.05; ^**^P<0.01. ‘*n*' represents the number of independent experiments.

β1-Integrin is a ubiquitous β subunit that pairs with at least 10 different α subunits. It has important roles in various cellular processes, including cell motility and growth.^26^ To determine whether neuron-released α-synuclein increases microglial cell motility, we performed the wound-healing assay. Rat primary microglial cultures were scratched before treatment with either LZCM or αSCM and incubated for 18 h. Treatment with αSCM significantly increased the rate of wound recovery (Figure 6), whereas the recovery after LZCM treatment did not differ significantly from that of Dulbecco's modified Eagle's medium treatment (Figure 6). When microglia were pre-incubated with either GRGDS or SDGRG, αSCM-induced acceleration of wound recovery was completely nullified by GRGDS, a functional β1-integrin antagonist (Figure 6), whereas pre-treatment with SDGRG had no effect (Figure 6). These results suggest that neuron-released α-synuclein increases microglial motility in a β1-integrin-dependent manner.

## Discussion

### Role of β1-integrin in α-synuclein-induced increase in microglial motility

The accumulation of activated microglia in disease-affected regions of the brain is a prominent pathological feature of LBdiseases.^27^ Microglia may be recruited to specific brain regions in response to various types of stimuli and transformed from the resting, ramified shape to the macrophage-like/ameboid phenotype.^28, 29^ The recruitment of microglia to specific brain regions and their activation may contribute to the progressive neurodegeneration.^28^ Therefore, how microglia are recruited to the disease-affected regions of the brain is one of the central questions in the study of neurodegenerative diseases.

Using gene expression profile analysis, we constructed a hypothetical intracellular signaling network that leads to increased cell motility in αSCM-treated microglia (Figure 2a). The network model strongly suggested a role for the integrin-signaling cascade in αSCM-induced cell motility. This model was functionally validated using peptide antagonists, showing that β1-integrin has critical roles in inducing this motility. In contrast, β1-integrin antagonists had no effect on cytokine gene induction or cell proliferation. These results suggest that β1-integrin signaling is specifically responsible for the migration of microglia to disease-affected regions of the brain.

### Integrins have roles in microglial activation induced by neurotoxic peptides

Integrins are heterodimeric transmembrane proteins (comprising one α and one β subunit) that are expressed on the surface of most cells.^30^ At present, 18 α and 8 β subunits have been identified, and 24 different α/β complexes have been demonstrated. Integrins are the major adhesion molecules involved in cell-to-cell and cell-to-extracellular matrix interactions, and they are critical for multiple cellular functions, including cell migration, phagocytosis and proliferation.^31^ Interestingly, multiple types of integrins interact with amyloid beta, mediating neuronal cell death and migroglial immune responses.^25, 32, 33, 34, 35, 36, 37, 38, 39, 40, 41, 42^ The amino-acid sequence of Aβ (RHD), which is structurally similar to the general integrin recognition sequence RGD, has been suggested as an integrin recognition site.^24, 25^ Our results indicate that the RHD peptide blocked αSCM-induced microglial responses and that there is a similar mode of interaction between α-synuclein and integrins. Consistent with our results, recent studies have shown the involvement of integrins in α-synuclein-induced cellular responses. Familial PD-linked mutant and wild-type α-synuclein induced αM-integrin activation via NADPH oxidase in microglia.^43^ Nitrated forms of α-synuclein have been shown to induce neuronal cell toxicity via interaction with α5β1-integrin.^44^ Integrins also interacted with a synthetic prion peptide; the functional inhibition of α5β1-integrin attenuated the activation of BV2 microglia induced by neurotoxic prion peptide PrP106-126.^45^ Thus, integrins may have important roles in neurodegenerative diseases through interactions with various disease-linked proteins.

Recent studies have suggested that receptors other than TLR2 and integrins act together with α-synuclein to cause microglial activation and/or clearance of extracellular α-synuclein. These receptors include TLR4 and CD36.^46, 47^ The conclusions of those studies were derived from experiments conducted with bacterially expressed recombinant α-synuclein. The roles of these proteins in microglial recruitment and activation need to be further validated with neuron-released α-synuclein.

### Microglial recruitment and activation by neuron-released α-synuclein

Neurons continuously release α-synuclein via unconventional exocytosis.^6^ The secretion of α-synuclein is minor in scale; however, it is regulated by protein folding stresses. Exocytosis of α-synuclein is increased by oxidative modifications of α-synuclein,^48, 49^ mitochondrial dysfunction^50^ and inhibition of autophagy.^51^ α-synuclein proteins released from ‘stressed' neurons were found to contain more oligomeric forms than the cytosolic proteins.^14, 50^ Neuron-released α-synuclein oligomers interacted with and activated TLR2 on the surface of microglia, thereby inducing proinflammatory responses.^14^ Considering these previous results together with our current study, we speculate that, in LB diseases, β1-integrin signaling is specifically responsible for the recruitment of microglia to the disease-affected brain regions, where neurons release relatively high levels of α-synuclein and its oligomeric forms. Furthermore, proinflammatory activation of the recruited microglia is mediated by the activation of TLR2 signaling. Our work suggests that both β1-integrin and neuron-released α-synuclein may have important roles in establishing local inflammation and may therefore be novel therapeutic targets for modifying neuroinflammation in LB diseases.

## Figures and Tables

**Table 1:** A list of primers and reaction conditions for RT-PCR analysis

*Gene (symbol)*	*Sequence (5′→3′)*	*Tm (°C)*	*Species*	*Product size (bp)*
*IL-1β*				
	F—TGAAGCAGCTATGGCAACTG	55	Rat	199
	R—TGCCTTCCTGAAGCTCTTGT			
				
*TNFα*				
	F—TGCCTCAGCCTCTTCTCATT	55.4	Rat	367
	R—TGTGGGTGAGGAGCACATAG			
				
*NOS2*				
	F—CACCTTGGAGTTCACCCAGT	60	Rat	170
	R—ACCACTCGTACTTGGGATGC			
				
*ICAM-1*				
	F—CAGGGTGCTTTCCTCAAAAG	55	Rat	249
	R—GGGCATGAGACTCCATTGTT			
				
*MMP-9*				
	F—GTCTTCCCCTTCGTCTTCCT	55	Rat	249
	R—AGGGGAGTCCTCGTGGTAGT			
				
*ITGa5*				
	F—AGGTGACGGGACTCAACAAC	60	Rat	151
	R—GGGCATTTCAGGACTTGTGT			
				
*SCIN*				
	F—ACCAGAGACGAGCTGACGAT	60	Rat	101
	R—GGCTCTTTGCCTTGAGACAC			
				
*Rac1*				
	F—TTTGAAAATGTCCGTGCAAA	60	Rat	306
	R—CAGCAGGCATTTTCTCTTCC			
				
*scin*				
	F—ACCAGAGACGAGCTGACGAT	60	Rat	101
	R—GGCTCTTTGCCTTGAGACAC			
				
*β-Actin*				
	F—TGTTGGCATAGAGGTCTTTACGG	60	Rat	278
	R—TGAGAGGGAAATCGTGCGTG			
				
*GAPDH*				
	F—ACCACAGTCCATGCCATCAC	60	Rat	452
	R—TCCACCACCCTGTTGCTGTA			
				
*IL-1 β*				
	F—CCGATGGGTTGTACCTTGTC	60	Mouse	284
	R—CGGACTCCGCAAAGTCTAAG			
				
*TNFα*				
	F—GACCTTCCAGGATGAGGACA	60	Mouse	283
	R—AGGCCACAGGTATTTTGTCG			
				
*β-actin*				
	F—TGTTACCAACTGGGACGACA	60	Mouse	391
	R—TCTCAGCTGTGGTGGTGAAG			

Abbreviations: IL, interleukin; RT-PCR, Reverse transcription polymerase chain reaction; TNF, tumor necrosis factor.

**Table 2:** A list of upregulated genes involved in integrin-mediated signaling pathways

*Cellular process (log2 scale)*	*Symbol*	*Fold change*
Integrin-mediated signaling pathway	Itgam	1.751581
	Pram1	1.299044
	Itga5	1.735116
	Vav1	1.179845
	Plek	1.527567
	Itgb2	2.349445
	Itgal	1.654326
